# AI Dependency, Academic Self-Efficacy, and Depressive Symptoms Among Medical Students: A Structural Equation Modeling Study in Saudi Arabia

**DOI:** 10.3390/healthcare14193291

**Published:** 2026-10-02

**Authors:** Saeed Asiri

**Affiliations:** Nursing Administration and Education Department, College of Nursing, King Saud University, Riyadh 11421, Saudi Arabia; saasiri@ksu.edu.sa

**Keywords:** artificial intelligence, AI dependency, academic self-efficacy, depressive symptoms, medical students, medical education, structural equation modeling, Saudi Arabia

## Abstract

**Background**: The rapid integration of artificial intelligence (AI) tools into medical education has opened up new possibilities for academic support. However, it has also raised concerns about over-dependence on AI. Students’ use of AI may have implications for students’ academic self-concept and psychological well-being, but the interrelationships among these constructs are not yet sufficiently understood. This study aimed to examine the association between AI dependency and academic self-efficacy and depressive symptoms among medical students in Saudi Arabia. **Methods**: We conducted a cross-sectional study among 207 medical students recruited from three public universities in Riyadh, Saudi Arabia. Data were collected anonymously using an online questionnaire distributed through QR-coded recruitment announcements. AI dependency was assessed using a 9-item subset of the AI Dependence Scale, academic self-efficacy using the 5-item General Academic Self-Efficacy Scale, and depressive symptoms using the two depression items of the PHQ-4. AI dependency and academic self-efficacy were modeled as latent constructs in a structural equation model. The model examined the direct association between AI dependency and depressive symptoms and the indirect associational pathway through academic self-efficacy. **Results**: The mean AI dependency score was 2.83 (SD = 0.78), while the mean academic self-efficacy score was 3.87 (SD = 1.07) and the mean depressive symptom score was 2.05 (SD = 1.42). AI dependency was positively associated with academic self-efficacy (β = 0.298, 95% CI 0.157–0.439, *p* < 0.001). Academic self-efficacy was inversely associated with depressive symptoms (β = −0.212, 95% CI −0.382–0.041, *p* = 0.016). AI dependency showed a strong positive direct association with depressive symptoms (β = 0.608, 95% CI 0.491–0.725, *p* < 0.001). The indirect association through academic self-efficacy was negative (β = −0.063, 95% CI −0.124–0.002, *p* = 0.052), while the total association remained positive and significant (β = 0.545, 95% CI 0.409–0.671, *p* < 0.001). The model explained 49% of the variance in academic self-efficacy and 37% of the variance in depressive symptoms. **Conclusions**: Greater AI dependency was associated with higher academic self-efficacy but also with substantially higher depressive symptom levels among medical students. Academic self-efficacy was inversely associated with depressive symptoms, although evidence for an indirect pathway between AI dependency and depressive symptoms was limited. Our findings highlight the importance of distinguishing productive AI use from excessive dependence. This supports the need for responsible AI integration alongside attention to medical students’ psychological well-being.

## 1. Introduction

In recent years, generative artificial intelligence (GenAI) has rapidly become part of university learning [1]. Large language models and related AI tools can generate explanations, summarize information, support writing, answer questions, and assist with problem solving. In medical education, these capabilities are particularly relevant because students routinely manage large volumes of information and increasingly use digital tools to support learning and research. A study among medical students found that 93.2% of them had used AI applications, with ChatGPT among the most frequently used tools [2]. Similarly, a 2026 study of Saudi medical students reported that 97.6% had previously used ChatGPT, with most students describing it as useful for understanding medical concepts and summarizing research [3].

The educational value of GenAI, however, depends on how students use it. AI can function as a learning aid that supports exploration, feedback, and access to information, but extensive reliance may change how students approach academic tasks [4]. However, it becomes concerning regarding the delegation of cognitive work to AI, including independent reasoning, problem solving, and critical evaluation. The recent development of the AI Dependence Scale for university students reflects growing recognition that AI dependence is distinct from simple frequency of use [5]. Wu et al. conceptualized AI dependence as a multidimensional construct involving emotional dependence, functional dependence, cognitive dependence, and loss of control [6]. Their validation study emphasized that dependence reflects the extent to which students become reliant on AI for academic functioning and cognitive processes.

This difference is specifically important in medical education. High AI use does not necessarily indicate problematic dependence. Students may use AI frequently while continuing to verify its responses, exercise independent judgment, and critically reflect on its limitations. Conversely, dependence may occur even with less frequent use when students experience difficulty completing academic tasks without AI. Recent research on AI-specific critical thinking similarly suggests that verification, motivation to understand AI, and reflection represent dimensions of engagement that are distinct from AI dependency [7]. Therefore, daily AI-use duration and AI dependency may capture different aspects of AI engagement and should be considered separately. A medical student may use an AI system frequently to clarify a difficult concept while continuing to reason independently and verify the information [8]. In contrast, dependence may arise when students experience difficulty completing academic tasks without AI, increasingly delegate thinking or writing to the system, feel uncomfortable when AI is unavailable, or struggle to reduce their use [9,10]. In medical study, on the other hand, students must progressively develop independent reasoning, judgment, and confidence before entering clinical practice. A recent systematic review among Saudi medical students indicates widespread AI adoption alongside concerns about accuracy, ethics, privacy, originality, and potential effects on critical thinking [11].

The psychological consequences of this pattern remain less well understood. Most previous studies on AI in medical education have focused on awareness, attitudes, readiness, perceived usefulness, and patterns of use. For example, recent Saudi studies have shown high levels of AI awareness and generally favorable attitudes among medical students [3]. They also identified concerns about reliability, ethics, privacy, and over-reliance. A systematic review and meta-analysis of medical undergraduates similarly found that students generally demonstrated moderate AI readiness and AI-related anxiety [12].

One potentially important psychological construct in this regard is academic self-efficacy. Academic self-efficacy is students’ belief in their ability to meet academic demands and complete academic tasks. It is highly associated with motivation, engagement, performance and psychological well-being. Van Zyl et al. also showed that the five-item General Academic Self-Efficacy Scale is a brief, reliable measure of students’ global academic self-efficacy [13]. Academic self-efficacy might be especially relevant to AI dependence, as the two constructs may interact in both a complementary and competitive manner. Students with high academic self-efficacy may use AI strategically because they see it as a learning supplement, not as a substitute for their own abilities. Conversely, students who are unsure of their academic ability may turn to AI to alleviate the difficulty or uncertainty of academic tasks. A survey of 300 university students revealed connections between academic self-efficacy, academic stress, performance expectations, and problematic AI use [14].

Academic self-efficacy may be relevant to this mental health burden because students’ beliefs about their ability to manage academic demands may influence the way they respond to challenges [15]. Studies that have specifically focused on medical students also find self-efficacy to be an important motivational construct, but past studies have used varied definitions and measurement methods. A critical review of 74 studies showed significant heterogeneity in the conceptualization and measurement of self-efficacy in medical students and emphasized the necessity of using theoretically rational and psychometrically valid measures [16].

The association between AI dependence, academic self-efficacy, and depressive symptoms may involve multiple, potentially reciprocal pathways. Students with lower self-efficacy may rely more on AI when facing academic difficulties. Conversely, repeated AI-assisted academic activity may be associated with students’ perceived academic capability by providing immediate explanations, feedback, and support. Thus, AI dependence may coexist with higher perceived self-efficacy without necessarily indicating greater independent academic competence. Academic self-efficacy may also be associated with fewer depressive symptoms because students who feel more capable of managing academic demands may experience less psychological distress. We therefore examined AI dependency, academic self-efficacy, depressive symptoms as an associational pathway, while recognizing that the reverse direction between AI dependency and self-efficacy is also plausible. Given the cross-sectional design, this pathway was treated as exploratory rather than causal. The study focused specifically on medical students in Saudi Arabia and examining AI dependence as a psychological and behavioral construct rather than simply measuring AI exposure or frequency of use. We examined whether AI dependency was associated with academic self-efficacy and depressive symptoms, and whether academic self-efficacy statistically accounted for part of the association between AI dependency and depressive symptoms. The directional pathway from AI dependency to academic self-efficacy was considered exploratory given the cross-sectional design and the possibility of reverse directionality.

## 2. Methods

### 2.1. Study Design and Setting

In this study, a cross-sectional study design was used. The targeted population comprised medical students enrolled at three public universities in Riyadh, Saudi Arabia. We performed data collection between 20 May and 15 June 2026 using an anonymous, self-administered online questionnaire. Our study specifically focused on medical students to examine the association between dependence on artificial intelligence (AI), academic self-efficacy, and depressive symptoms within a population that frequently encounters demanding academic and clinical learning tasks.

The data collection was performed electronically using Google Forms. Recruitment announcements were distributed through the participating universities. We provided a QR code linking directly to the online questionnaire displayed on university announcement boards. Participation was voluntary, and students completed the questionnaire independently. The cross-sectional design allowed assessment of the relationships among AI dependency, academic self-efficacy, and depressive symptoms at the time of data collection.

### 2.2. Participants and Eligibility

The target population comprised undergraduate and postgraduate medical students enrolled at the participating public universities in Riyadh. Participants were eligible if they were currently enrolled as medical students and completed the relevant sections of the online questionnaire. Responses that did not contain sufficient information for calculation of the primary study measures were excluded from the corresponding analyses.

A total of 232 students responded to the questionnaire. Of these, 25 responses were excluded because most of these respondents did not provide sufficient responses to the validated instruments required for the primary analysis. The final sample included 207 medical students, and these 207 participants were included in the SEM analysis. The sample included students across different years of medical education, allowing adjustment for stage of training in the multivariable analyses.

### 2.3. Measures and Variables

#### 2.3.1. AI Dependency

To evaluate AI dependency, we used a 9-item subset of the AI Dependence Scale (AIDep-22) developed and validated by Wu et al. [6]. The original instrument contains 22 items assessing dependence on AI among university students. For our study, nine items were selected to represent three key domains of AI dependence: cognitive dependence, functional dependence, and loss of control, with three items representing each domain.

The selected nine items assessed difficulties completing academic tasks without AI, reliance on AI for organizing thoughts or producing written content, discomfort when AI tools were unavailable, perceived weakening of independent critical thinking, reduced confidence without AI confirmation, preference for AI-generated explanations over independent effort, difficulty reducing AI use, anxiety or stress when completing tasks without AI, and AI use exceeding the user’s original intention. Items were assessed using a 5-point Likert scale ranging from 1 = strongly disagree to 5 = strongly agree. We calculated the mean of the nine items to get the overall AI dependency score. Higher scores indicated greater AI dependency. In our study, the scale demonstrated good internal consistency, with Cronbach’s α = 0.837 and McDonald’s ω = 0.940.

The nine items were selected from the original AIDep-22 based on their conceptual relevance to cognitive dependence, functional dependence, and loss of control, with three items representing each domain. The questionnaire was administered in English. The nine items were modeled as indicators of a single latent AI-dependency construct because the study focused on overall problematic reliance on AI rather than separate domain-specific effects. This specification was based on the conceptualization of the selected domains as related components of AI dependency and was evaluated using the measurement model. We therefore modeled AI dependency as a single latent construct in the SEM, with the nine items serving as its observed indicators. This specification was further supported by the standardized factor loadings, which ranged from 0.633 to 0.881.

#### 2.3.2. Academic Self-Efficacy

The 5-item General Academic Self-Efficacy Scale (GASE-5) developed and evaluated by van Zyl et al. was used to evaluate academic self-efficacy [13]. The scale assesses students’ perceived confidence in their ability to achieve academic success, master difficult academic material through effort, meet university demands, overcome academic difficulties, and succeed in their academic career.

Each item was rated on a 5-point response scale ranging from 1 = not at all true to 5 = completely true. Responses were averaged to produce an overall academic self-efficacy score. Higher scores indicate greater perceived academic self-efficacy. The scale showed excellent internal consistency in this study, with Cronbach’s α = 0.943 and McDonald’s ω = 0.945.

#### 2.3.3. Depressive Symptoms

Depressive symptoms were assessed using the depression component of the PHQ-4. It consists of two items assessing depressed mood and diminished interest or pleasure. Each item was scored from 0 = not at all to 3 = nearly every day. This resulted in a depressive symptom score ranging from 0 to 6, with higher scores indicating greater depressive symptom burden.

The study used the two depression items to calculate a symptom score rather than applying a clinical diagnosis. The PHQ-4 is a brief screening measure and was therefore used to quantify the presence and severity of depressive symptoms in the study population rather than to establish a psychiatric diagnosis [17].

#### 2.3.4. AI Use and Sociodemographic Characteristics

Participants reported their average daily duration of AI tool use and the AI tool they used most frequently for their studies. Daily AI use was originally recorded across six categories: no AI use, less than 30 min, 30 min to 1 h, 1–2 h, 2–4 h, and more than 4 h. For analysis, the first two duration categories below 1 h were combined, resulting in four substantive use categories plus a no-use category: no AI use, <1 h, 1–2 h, 2–4 h, and >4 h.

We also collected data on gender, age group, year of study, and GPA. Age was categorized as 18–20, 21–23, and ≥24 years, with the two oldest original categories combined. Year of study was grouped into Years 1–2, Year 3, Year 4, and Year 5+/postgraduate. GPA was grouped into <3.0, 3.0–3.49, and 3.5–4.0.

### 2.4. Statistical Analysis

Descriptive statistics were calculated for participant characteristics, AI-use patterns, AI dependency, academic self-efficacy, and depressive symptoms. Categorical variables were summarized using frequencies and percentages, while continuous scale scores were summarized using means and standard deviations.

Internal consistency of the AI dependency and academic self-efficacy scales was assessed using Cronbach’s alpha and McDonald’s omega. Higher values indicated greater internal consistency.

The primary analysis used structural equation modeling (SEM) to examine the hypothesized relationships among AI dependency, academic self-efficacy, and depressive symptoms. AI dependency and academic self-efficacy were modeled as latent variables, using their respective nine and five questionnaire items as indicators. Depressive symptoms were modeled as an observed continuous outcome based on the PHQ-2 component of the PHQ-4.

The structural model specified academic self-efficacy as an associational mediator of the association between AI dependency and depressive symptoms. Specifically, the model estimated the pathway from AI dependency to academic self-efficacy. The associational pathway was from academic self-efficacy to depressive symptoms, and the direct association from AI dependency to depressive symptoms. The indirect association was estimated as the product of the two constituent paths. The total effect was calculated as the sum of the direct and indirect effects. The structural model was estimated using maximum likelihood estimation with full-information maximum likelihood (FIML) for any item-level missing data among participants retained in the SEM analysis. Bootstrap standard errors and 95% confidence intervals were obtained using 5000 bootstrap resamples. Standardized regression coefficients (β) and corresponding 95% confidence intervals were reported.

The structural model was adjusted for gender, age group, year of study, and daily AI-use duration. Categorical covariates were recoded into indicator variables before inclusion in the SEM, with male students, students aged 21–23 years, Year 3 students, and students reporting <1 h of daily AI use serving as the respective reference groups.

Model fit was evaluated using the chi-square statistic, comparative fit index (CFI), Tucker–Lewis index (TLI), root mean square error of approximation (RMSEA) with its 90% confidence interval, and standardized root mean square residual (SRMR). The Akaike information criterion (AIC) and Bayesian information criterion (BIC) were also reported as information criteria. Standardized regression coefficients were reported with 95% confidence intervals.

The proportion of variance explained in each endogenous variable was assessed using R^2^. Statistical inference for the associational pathway was based on the estimated indirect effect and its confidence interval. Because the study used cross-sectional data, all associations (pathways) were interpreted as non-causal associations. The mediation analysis was also interpreted as an examination of statistical pathways (associations) rather than evidence of temporal or causal mediation.

All analyses were conducted using R statistical software (version 4.2.1) [18]. We used the *lavaan* package (version 0.6-21) for all SEM analyses,

## 3. Results

Table 1 shows that, among the 207 medical students, 56.0% were male and 44.0% were female. Most participants were aged 21–23 years (62.8%). About 22.2% were aged 24 years or older, and 15.0% were 18–20 years old. Half of the participants were in Year 3 (50.2%), while 22.2% were in Year 4, 16.4% were in Years 1–2, and 11.1% were in Year 5 or postgraduate studies.

The results showed that approximately one-third of students had a GPA below 3.0 (33.3%), 36.7% had a GPA of 3.0–3.49, and 30.0% had a GPA of 3.5–4.0. AI use was common, with 47.3% reporting less than 1 h of daily use, 15.0% reporting 1–2 h, 13.0% reporting 2–4 h, and 21.7% reporting more than 4 h. Only 2.9% reported no AI use. Gemini (45.9%) and ChatGPT (41.1%) were the most commonly used AI tools.

The mean AI dependency score was 2.83 (SD = 0.78), while the mean academic self-efficacy score was 3.87 (SD = 1.07). The mean depressive symptom score was 2.05 (SD = 1.42).

The SEM summary results are provided in Table 2. We found that AI dependency was positively associated with academic self-efficacy (β = 0.298, 95% CI 0.157–0.439, *p* < 0.001), whereas academic self-efficacy was inversely associated with depressive symptoms (β = −0.212, 95% CI −0.382–0.041, *p* = 0.016). AI dependency also showed a strong positive direct association with depressive symptoms (β = 0.608, 95% CI 0.491–0.725, *p* < 0.001). The indirect association between AI dependency and depressive symptoms through academic self-efficacy was negative (β = −0.063). The negative indirect association did not reach statistical significance, indicating that the data did not provide evidence for an indirect association between AI dependency and depressive symptoms through academic self-efficacy. The total association between AI dependency and depressive symptoms remained positive and significant (β = 0.545, 95% CI 0.409–0.671, *p* < 0.001). Our findings indicate that greater AI dependency was associated with higher scores for depressive symptoms, while academic self-efficacy showed an inverse association with depressive symptoms. However, the data provide limited evidence that academic self-efficacy may account for part of the association between AI dependency and depressive symptoms.

The standardized factor loadings were consistently high, ranging from 0.633 to 0.881 for AI dependency and 0.770 to 0.924 for academic self-efficacy (Supplementary Table S1). This supports the validity of the specified measurement model.

We also evaluated the association of other covariates with academic self-efficacy and with depression symptoms (Supplementary Table S2). We found that higher age and greater daily AI use were associated with lower academic self-efficacy. Students aged ≥24 years reported lower academic self-efficacy than those aged 21–23 years (β = −0.175, 95% CI −0.308–0.050). Compared with students using AI for <1 h per day, lower self-efficacy was also observed among those reporting 1–2 h (β = −0.173, 95% CI −0.306–0.039), 2–4 h (β = −0.247, 95% CI −0.387–0.122), and >4 h of daily AI use (β = −0.256, 95% CI −0.379–0.085). Lower self-efficacy was observed among female participants. Year of study showed a relatively small association with academic self-efficacy.

We also assessed model diagnosis for multivariate regression and did not find model violations (Supplementary Figure S1).

In contrast, none of the examined demographic or AI-use covariates showed a clear association with depressive symptoms. The results indicate that the covariate pattern was more pronounced for academic self-efficacy than for depressive symptoms, particularly for age and daily AI-use duration.

The final latent-variable model showed moderate overall fit to the data (Table 3). The model had χ^2^ = 458.02 with 216 degrees of freedom, *p* < 0.001. The CFI was 0.93 and TLI was 0.91, while RMSEA was 0.074 (90% CI 0.064–0.083). SRMR was 0.102. Overall, the model showed acceptable approximate fit according to CFI, TLI, and RMSEA, although the SRMR indicated some residual model misfit.

The model explained 49% of the variance in academic self-efficacy and 37% of the variance in depressive symptoms. This indicates that the predictors included in the model accounted for a substantial proportion of variability in both endogenous outcomes.

**Figure 1:**
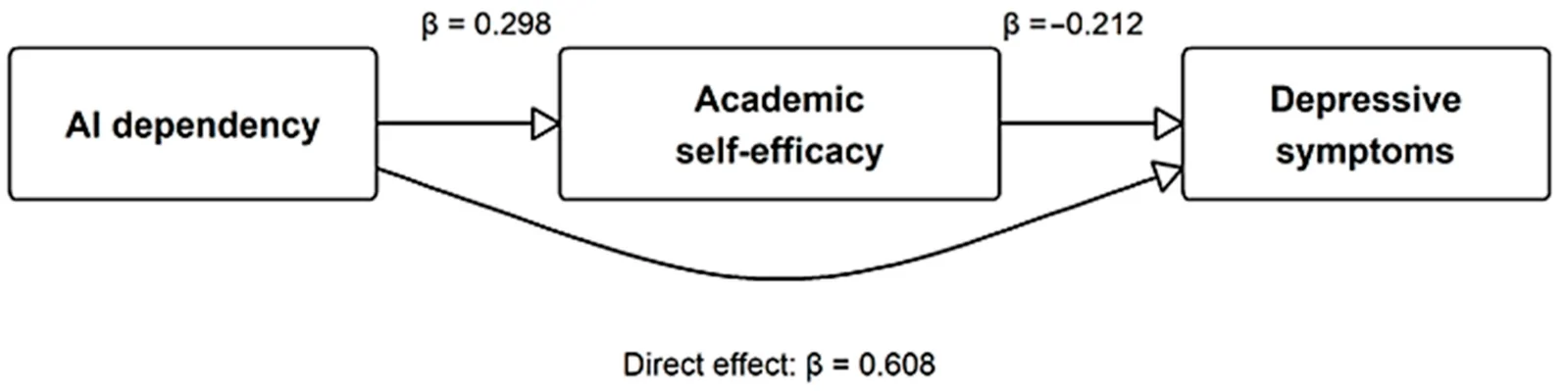
Latent mediation model of the associations between AI dependency, academic self-efficacy, and depressive symptoms among medical students.

We further visualized the direct and indirect associations in SEM (Figure 1). The model showed a positive association between AI dependency and academic self-efficacy (β = 0.298), while academic self-efficacy was inversely associated with depressive symptoms (β = −0.212). AI dependency also showed a strong positive direct association with depressive symptoms (β = 0.608). The resulting indirect association through academic self-efficacy was negative (β = −0.063), whereas the total association between AI dependency and depressive symptoms remained positive (β = 0.545).

This pattern indicates that greater AI dependency was associated with higher academic self-efficacy, which was in turn associated with fewer depressive symptoms, while AI dependency simultaneously showed a substantial positive direct association with depressive symptoms. This pattern suggests a potentially competing indirect pathway through academic self-efficacy. However, because the study was cross-sectional, these pathways should be interpreted as associations rather than causal effects.

## 4. Discussion

### 4.1. Main Findings of This Study

Our study examined the associations among AI dependency, academic self-efficacy, and depressive symptoms in 207 medical students in Saudi Arabia using a latent-variable structural equation model. We found that AI dependency was positively associated with academic self-efficacy. The finding also showed that higher academic self-efficacy was associated with fewer depressive symptoms. AI dependency also showed a strong positive direct association with depressive symptoms even after accounting for academic self-efficacy and the prespecified covariates. The indirect association through academic self-efficacy was negative but did not reach statistical significance. Our findings suggest that AI dependency may coexist with greater perceived academic confidence while also being associated with poorer psychological well-being.

### 4.2. Comparison to Existing Studies

While our findings showed a positive association between AI dependency and academic self-efficacy, other evidence suggests that the relationship may be more complex. For example, a study of university students reported a negative correlation between academic self-efficacy and AI dependence [19]. A study of 300 university students found that academic self-efficacy was related to AI dependency through academic stress and performance expectations, indicating that academic confidence, stress, expectations, and AI reliance may interact rather than follow a simple linear pattern [14]. More recent work has also examined academic self-efficacy as a psychological pathway involving GenAI dependence, academic stress, and perceived usefulness and found a strong association [20].

Several explanations may be possible for the positive association observed in our findings. Students may experience AI as an effective source of immediate academic support. When AI provides rapid explanations, examples, summaries, or assistance with difficult tasks, successful AI-assisted performance may reinforce students’ perceptions that they can manage academic demands. In this situation, dependence and confidence can coexist. Students may feel academically capable precisely because AI reduces the perceived difficulty of academic tasks. On the other hand, academic self-efficacy and actual independent academic capability are conceptually different constructs. Self-efficacy reflects perceived capability rather than objectively measured competence. A student may report high confidence in succeeding academically while relying extensively on AI to complete or support academic tasks. Therefore, the positive association should not be interpreted as evidence that greater AI dependency improves students’ independent academic abilities.

The conceptualization of AI dependency may also help explain the observed findings. Recent scale-development work has described GenAI dependency as involving cognitive preoccupation, negative consequences, and withdrawal, emphasizing that dependency reflects problematic reliance rather than simply frequent AI use [21]. In our study, the nine-item measure similarly focused on cognitive dependence, functional reliance, and loss of control. This includes difficulty completing tasks without AI, reliance on AI for organizing thoughts or writing, discomfort when AI is unavailable, and difficulty reducing AI use. The construct assessed here represents problematic reliance on AI for academic functioning rather than general AI exposure. The positive association with academic self-efficacy should therefore be interpreted cautiously and may reflect the distinction between perceived academic capability and independent academic functioning.

Our findings showed that AI dependency was strongly and positively associated with depressive symptoms. This association remained strong after accounting for academic self-efficacy, gender, age group, year of study, and daily AI-use duration. The finding suggests that the psychological implications of AI dependency extend beyond academic functioning. This means that dependence on AI may reflect broader difficulties with autonomous academic functioning. Our findings were consistent with previous studies. A study conducted in all 50 states of the USA showed that there was a modest association between AI use and depressive symptoms [22]. A recent study in 5748 Chinese college students also indicated that GenAI profiles were associated with anxiety and depression [23]. The findings should be interpreted cautiously, because the cross-sectional design cannot determine whether AI dependency contributes to depressive symptoms, whether students experiencing depressive symptoms are more likely to rely on AI, or whether both arise from common underlying factors such as academic stress. Reverse and reciprocal relationships are plausible. For example, students experiencing psychological distress may have reduced motivation or energy for independent academic work and may therefore turn more frequently to AI for assistance.

We found that academic self-efficacy was inversely associated with depressive symptoms. Students who reported greater confidence in their ability to manage academic demands tended to report fewer depressive symptoms. This finding is consistent with the broader literature linking students’ perceived academic competence with psychological well-being. A previous study showed that depression is negatively associated with academic self-efficacy [24]. Another study on 569 college students indicated that academic self-efficacy was strongly and inversely associated with depression [25]. The association observed in our study is particularly important as we focused on medical students. Medical students face sustained academic and performance demands, and depressive symptoms are common within this population [26,27,28]. Academic self-efficacy may contribute to how students perceive and respond to academic challenges because students who believe they can manage difficult tasks may experience fewer feelings of helplessness or failure. Our findings also provide an important context for interpreting the positive AI-dependency-to-self-efficacy associational pathway. Although AI dependency was associated with greater perceived academic self-efficacy, higher self-efficacy was associated with fewer depressive symptoms. Thus, the association between AI dependency and depressive symptoms cannot be adequately understood by considering academic confidence alone.

The indirect associational pathway from AI dependency to depressive symptoms through academic self-efficacy was negative, though the association was not significant. The inverse association indicates that the associational pathway from greater AI dependency to higher academic self-efficacy, followed by higher academic self-efficacy to fewer depressive symptoms, operated in the opposite direction to the direct association. Recent research supports the value of considering multiple psychological pathways rather than assuming a single mechanism. Studies have examined AI dependence in relation to academic stress, performance expectations, self-control, AI literacy, and other psychological variables [14]. A recent study has also reported associations between AI dependence, self-esteem, and psychological distress, although those findings cannot be directly equated with the present cross-sectional results [29]. In our findings, the relatively small indirect effect compared with the direct effect further suggests that academic self-efficacy alone does not adequately explain the relationship between AI dependency and depressive symptoms.

### 4.3. Strengths and Limitations

This study has several strengths. We focused specifically on medical students, a population for whom AI use has immediate relevance to academic and future professional practice. In our study, AI dependency and academic self-efficacy were modeled as latent constructs. This allows the analysis to account for measurement error and use multiple indicators of each construct. The study examined the association among AI dependency, academic self-efficacy, and depressive symptoms simultaneously rather than considering each association separately. Finally, we also adjusted for relevant demographic and academic characteristics, including daily AI-use duration.

Several limitations should be acknowledged. We used a cross-sectional design, which does not allow us to assess the temporal ordering or causality. The hypothesized mediation pathway should therefore be interpreted as a statistical association rather than evidence that AI dependency causes changes in academic self-efficacy or depressive symptoms. Longitudinal studies are needed to establish temporal relationships and determine whether changes in AI dependency precede changes in academic confidence or psychological symptoms. Our study relied on self-reported measures. Students may underreport or overreport their AI use, dependency, self-efficacy, or depressive symptoms. Participants were recruited from three public universities in Riyadh using an online questionnaire distributed through university announcement boards. Although this approach allowed recruitment across multiple institutions, it was not a probability-based sampling strategy. The findings may therefore not generalize to all medical students in Saudi Arabia. In this study, we used a nine-item subset of the AIDep-22 rather than the complete 22-item instrument. Although the selected items were intended to represent cognitive dependence, functional dependence, and loss of control and demonstrated strong internal consistency in the present sample, future studies should further examine the measurement invariance and construct validity of this abbreviated version in Saudi medical students. Finally, depressive symptoms were assessed using the two-item depression component of the PHQ-4. This provides a brief measure of depressive symptom burden but does not establish a clinical diagnosis of depression. The findings should therefore be interpreted in terms of depressive symptoms rather than depressive disorders.

## 5. Conclusions

Our study identified a complex association between AI dependency, academic self-efficacy, and depressive symptoms among medical students in Saudi Arabia. Greater AI dependency was associated with higher academic self-efficacy, while greater academic self-efficacy was associated with fewer depressive symptoms. At the same time, AI dependency showed a strong positive direct association with depressive symptoms. Our findings suggest that AI dependency should not be viewed simply as an indicator of poor academic confidence. Students may feel highly capable academically while simultaneously becoming substantially reliant on AI. At the same time, greater dependence may be associated with psychological difficulties through pathways that are not explained by academic self-efficacy. Future longitudinal research should examine these relationships over time and investigate additional mechanisms, particularly academic stress, self-control, AI literacy, perceived performance, and other dimensions of student well-being.

## Figures and Tables

**Table 1:** Characteristics of the medical student sample (N = 207).

Variable	Category	n (%)/Mean (SD)
Gender	Male	116 (56.0%)
	Female	91 (44.0%)
Age group	18–20	31 (15.0%)
	21–23	130 (62.8%)
	24 or older	46 (22.2%)
Year of study	Years 1–2	34 (16.4%)
	Year 3	104 (50.2%)
	Year 4	46 (22.2%)
	Year 5+/Postgraduate	23 (11.1%)
GPA	<3.0	69 (33.3%)
	3.0–3.49	76 (36.7%)
	3.5–4.0	62 (30.0%)
Daily AI use	No AI use	6 (2.9%)
	<1 h	98 (47.3%)
	1–2 h	31 (15.0%)
	2–4 h	27 (13.0%)
	>4 h	45 (21.7%)
Most-used AI tool	ChatGPT	85 (41.1%)
	Gemini	95 (45.9%)
	Other AI tools	21 (10.1%)
	No AI use	6 (2.9%)
AI dependency score	Mean (SD)	2.83 (0.78)
Academic self-efficacy score	Mean (SD)	3.87 (1.07)
Depressive symptoms	Mean (SD)	2.05 (1.42)

**Table 2:** Latent mediation model of the association between AI dependency and depressive symptoms.

Path	β (95% CI)	*p* Value
AI dependency → Academic self-efficacy	0.298 (0.157–0.439)	<0.001
Academic self-efficacy → Depressive symptoms	−0.212 (−0.382–0.041)	0.016
AI dependency → Depressive symptoms, direct effect	0.608 (0.491–0.725)	<0.001
AI dependency → Academic self-efficacy → Depressive symptoms, indirect effect	−0.063 (−0.124–0.002)	0.052
AI dependency → Depressive symptoms, total effect	0.545 (0.409–0.671)	<0.001

**Table 3:** Model fit indices and explained variance for the final latent mediation model.

Fit Index	Value	Endogenous Variable	R^2^
χ^2^	458.02	Academic self-efficacy	0.49
df	216	Depressive symptoms	0.37
*p* value	<0.001		
CFI	0.93		
TLI	0.91		
RMSEA	0.074		
90% CI for RMSEA	0.064–0.083		
SRMR	0.102		
AIC	7677.07		
BIC	7907.03		

## Data Availability

The data presented in this study are available on request from the corresponding author. The data are not publicly available due to privacy and ethical restrictions.

## Supplementary Materials

The following supporting information can be downloaded at: https://www.mdpi.com/article/10.3390/healthcare14193291/s1, Figure S1. Regression diagnostic plots for the multivariable linear regression model of academic self-efficacy; Table S1. Standardized factor loadings of the latent constructs; Table S2. Covariates associated with academic self-efficacy.
