# Supplementary material for: AI Dependency, Academic Self-Efficacy, and Depressive Symptoms Among Medical Students: A Structural Equation Modeling Study in Saudi Arabia
Source: Healthcare (Basel). 2026 Oct 2;14(19):3291. doi: 10.3390/healthcare14193291 (PMC13665285; doi:10.3390/healthcare14193291)
Supplement: Supplementary file 1 [file healthcare-14-03291-s001.zip › healthcare-4565409-supplementary.pdf]

Supplementary Materials

AI Dependency, Academic Self-Efficacy, and Depressive Symptoms Among Medical Students: A Structural Equation Modeling Study in Saudi Arabia

Saeed Asiri

Nursing Administration and Education Department, College of Nursing, King Saud University, Riyadh 11421, Saudi Arabia; saasiri@ksu.edu.sa

Table S1. Standardized factor loadings of the latent constructs

Latent construct	Item	Standardized loading
**AI dependency**	AI1	0.881
	AI2	0.779
	AI3	0.858
	AI4	0.633
	AI5	0.791
	AI6	0.778
	AI7	0.835
	AI8	0.807
	AI9	0.858
**Academic self-efficacy**	SE1	0.770
	SE2	0.895
	SE3	0.914
	SE4	0.924
	SE5	0.924

Table S2. Covariates associated with academic self-efficacy

Predictor	Academic self-efficacy, β (95% CI)	Depressive symptoms, β (95% CI)
Female vs male	-0.274 (-0.457--0.078)	-0.022 (-0.209-0.164)
Age 18-20 vs 21-23	-0.035 (-0.151-0.097)	-0.059 (-0.198-0.085)
Age ≥24 vs 21-23	-0.175 (-0.308--0.050)	-0.040 (-0.154-0.088)
Year 1-2 vs Year 3	-0.083 (-0.225-0.047)	0.113 (-0.044-0.244)
Year 4 vs Year 3	0.024 (-0.087-0.135)	-0.030 (-0.171-0.097)
Year 5+ / postgraduate vs Year 3	-0.021 (-0.161-0.123)	0.087 (-0.045-0.221)
No AI use vs <1 hour	0.028 (-0.121-0.157)	0.018 (-0.052-0.091)
1-2 hours vs <1 hour	-0.173 (-0.306--0.039)	-0.028 (-0.198-0.135)
2-4 hours vs <1 hour	-0.247 (-0.387--0.122)	0.037 (-0.085-0.167)
>4 hours vs <1 hour	-0.256 (-0.379--0.085)	0.003 (-0.108-0.103)

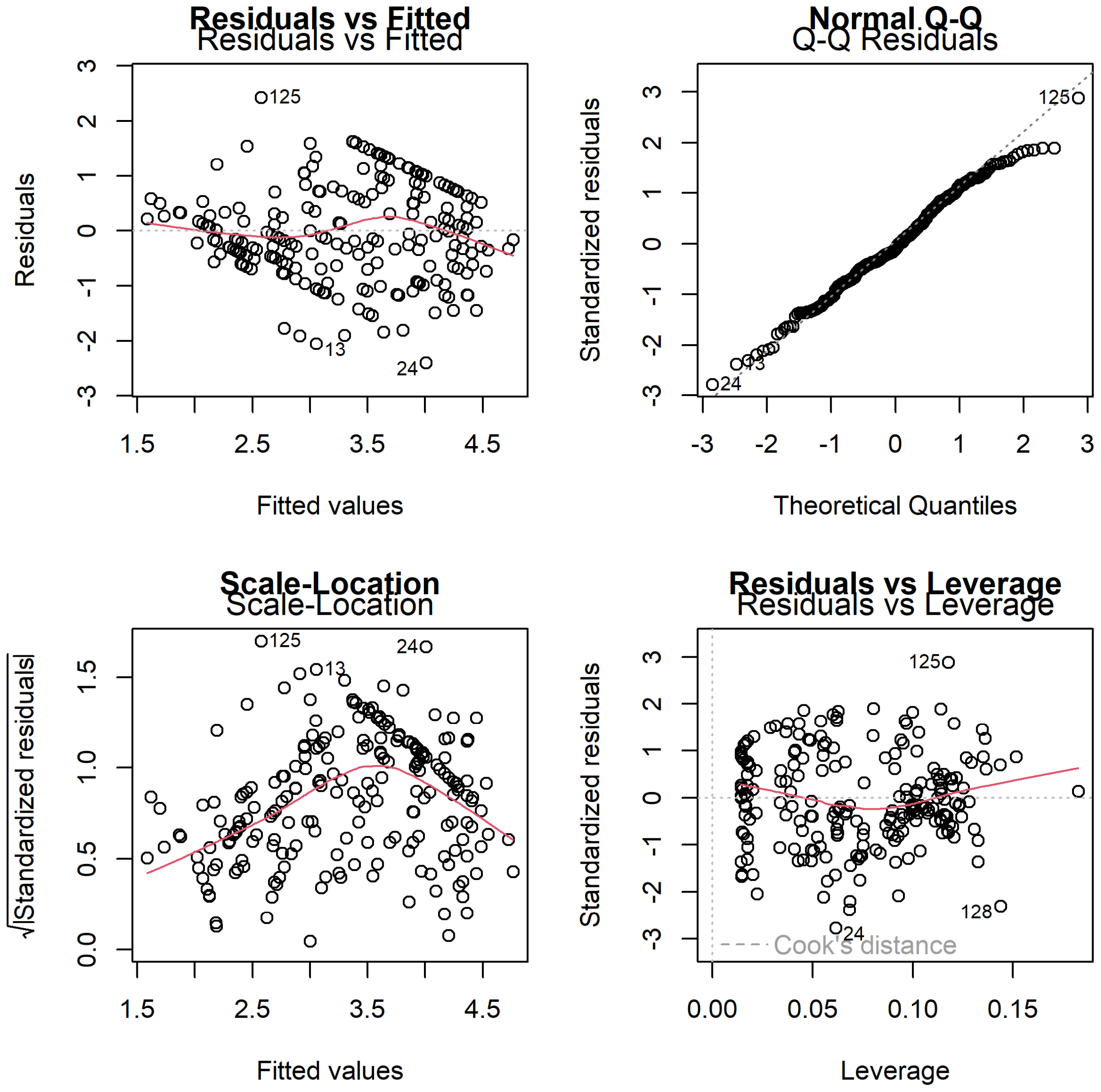

Figure S1. Regression diagnostic plots for the multivariable linear regression model of academic self-efficacy
